# Wastewater-Based Surveillance of Substances of Potential Abuse

**DOI:** 10.23889/ijpds.v9i5.2584

**Published:** 2024-09-18

**Authors:** Danielle Southern, Monty Ghosh, Megan Harmon, Darina Kuzma, Aleshia Kormendi, Kayla Moffett, Paul Westlund, Casey Hubert, Michael Parkins, Tyler Williamson

**Affiliations:** 1 Centre for Health Informatics, University of Calgary; 2 Alberta Health Services; 3 University of Calgary; 4 CEC Analytics

## Objectives

Amid a drug poisoning epidemic, UCalgary adapted its comprehensive wastewater monitoring program to track illicit drug use. The joint initiative between the academic community and Alberta municipalities aims to provide early warning information on changes in the drug supply to healthcare workers, policymakers, and people with substance use disorders (pwSUD).

## Approach

The logistic network, first used for SARS-CoV-2 monitoring, has evolved to track 48 substances linked to substance use. These include the parent drugs (e.g., cocaine, methamphetamines, various opiates) and their metabolites. The comprehensive nature of this tracking provides a nuanced understanding of trends. Collected up to 3X-weekly across various locations in Alberta including municipal wastewater treatment plants, entire neighbourhoods, and shelters, the data reveals geographical and temporal patterns in substance use. Findings were cross validated with Alberta’s substance use surveillance system to predict trends in wastewater, correlating with emergency and inpatient admissions and overdose deaths.

## Results

The research uncovered noteworthy trends. Intermittent, alarming, spikes in carfentanyl, benzodiazepines, and xylazine in wastewater were observed during periods of elevated Emergency Medical Services (EMS) responses to opioid-related incidents in the year 2023. This correlation underscores the real-world impact of the identified substance trends, emphasizing the urgency for targeted interventions to address the escalating opioid crisis. Further work to create a dashboard with additional data sources has begun. 

## Conclusions

Timely data on the dynamic drug supply is crucial in addressing the ongoing drug poisoning crisis. The team is exploring ways to share information with emergency responders, healthcare providers, government officials, and pwSUD.

